# Clinical analysis of ultrasound-guided laparoscopic microwave ablation in the treatment of early HCC with severe thrombocytopenia

**DOI:** 10.3389/fsurg.2026.1836697

**Published:** 2026-10-30

**Authors:** Heyue Zhang, Weiming Zhang, Wei Wu, Wenxin Li, Jin Xu, Xudong Zhu

**Affiliations:** 1 Liaoning Provincial Key Laboratory of Precision Medicine for Malignant Tumors, Liaoning Cancer Hospital & Institute, Cancer Hospital of Dalian University of Technology, Shenyang, Liaoning, China; 2 Department of First General Surgery, The Sixth People’s Hospital of Shenyang, Shenyang, Liaoning, China; 3 Hepatobiliary and Pancreatic Surgery Center, the Sixth People’s Hospital of Shenyang, Shenyang, Liaoning, China; 4 Department of Hepatopancreatobiliary Surgery, Cancer Hospital of Dalian University of Technology, Liaoning Cancer Hospital & Institute, Shenyang, Liaoning, China

**Keywords:** early HCC, laparoscopy, microwave ablation, severe thrombocytopenia, treatment

## Abstract

**Background:**

Patients with hepatocellular carcinoma (HCC) frequently present with cirrhosis accompanied by thrombocytopenia, and severe thrombocytopenia (STP) represents a major therapeutic challenge in this population. The present study was designed to evaluate the safety and efficacy of ultrasound-guided laparoscopic microwave ablation for the management of early HCC with STP.

**Methods:**

Clinical data from patients with early HCC who underwent ultrasound-guided laparoscopic microwave ablation at the Department of General Surgery I, Sixth People's Hospital of Shenyang between February 2023 and February 2024 were retrospectively analyzed. Patients were categorized into a study group (PLT < 50 × 10^9^/L) and a control group (PLT > 150 × 10^9^/L). Follow-up was continued until February 1, 2025, and postoperative recovery outcomes and disease-free survival were compared between groups. Propensity score matching (PSM) was applied to minimize baseline bias. A total of 49 patients with STP were included in the study group, while 48 patients with normal platelet counts were included in the control group. After 1:1 PSM, 22 patients were retained in each group.

**Results:**

The study group exhibited greater intraoperative blood loss than the control group both before and after matching. However, no statistically significant differences were observed between the two groups with respect to operative duration, length of postoperative hospitalization, incidence of complications, or disease-free survival.

**Conclusion:**

Ultrasound-guided laparoscopic microwave ablation is a safe and feasible therapeutic option for patients with early HCC and STP, demonstrating favorable short-term outcomes.

## 1 Introduction

Thrombocytopenia (TP) is internationally defined as a platelet count (PLT) below 150 × 10^9^/L, with severe thrombocytopenia (STP) defined as a PLT below 50 × 10^9^/L (1). Clinically, TP is observed in approximately 80% of patients with cirrhosis (2). In addition, most cases of HCC [Barcelona Clinic Liver Cancer (BCLC) stage 0/A] arise secondary to cirrhosis (3). At present, radical treatment options for HCC include partial hepatectomy, liver transplantation, and liver tumor ablation. In early HCC, partial hepatectomy demonstrates outcomes comparable to those of local ablation (4). For HCC with Child-Pugh grade A liver function, surgical resection remains the preferred therapeutic approach. However, the severity of cirrhosis and Child-Pugh classification are generally positively associated with the extent of hypersplenism. Patients with STP seldom exhibit grade A liver function and are exposed to an increased risk of perioperative hemorrhage and postoperative liver failure (5–7). Liver transplantation can address cirrhosis, hypersplenism, and HCC simultaneously; however, widespread application is limited by high costs and donor scarcity. When these considerations are taken into account, local ablation represents a relatively safe radical treatment strategy for patients with STP (8).

A PLT < 50 × 10^9^/L is commonly regarded as a relative contraindication for liver biopsy, interventional therapy for HCC, ablation, surgical resection, and targeted immunotherapy. Previous studies have demonstrated that PLT below 50 × 10^9^/L is associated with a threefold increase in the risk of spontaneous bleeding (9, 10). Nevertheless, except for invasive approaches such as splenectomy and splenic embolization, which can rapidly elevate and stabilize platelet levels, pharmacologic therapy or platelet transfusion is unlikely to achieve or sustain PLT within an optimal range (11, 12). Concomitant splenectomy during HCC treatment or preoperative splenic embolization has been proposed to reduce postoperative hemorrhagic risk or elevate PLT (12). However, such strategies may also induce portal vein thrombosis, thereby aggravate portal hypertension and increase the incidence of complications, including intra-abdominal infection (13). In this patient population, achieving an appropriate balance between effective tumor eradication and perioperative bleeding risk remains a major clinical challenge.

Percutaneous ultrasound-guided ablation is limited in patients with severe thrombocytopenia and cirrhosis: diaphragmatic lesions may be obscured by pulmonary air, tumors near the gallbladder, gastrointestinal tract, or major vessels are difficult to protect, and superficial lesions carry a risk of tract seeding (14, 15). Laparoscopic ultrasound-guided microwave ablation (MWA) may overcome these limitations through artificial ascites and direct visualization of needle placement, ablation, and tract coagulation, improving local hemostasis (16). Compared with radiofrequency ablation, MWA offers a larger ablation zone, shorter ablation time, and reduced heat-sink effect (14). However, evidence on laparoscopic MWA in early HCC with severe thrombocytopenia is limited, and its impact on perioperative recovery and disease-free survival versus normal platelet counts remains unclear (17).

Accordingly, the present study retrospectively evaluated the safety and short-term efficacy of ultrasound-guided laparoscopic MWA in BCLC stage 0/A HCC patients with STP and compared outcomes with those of patients with normal platelet counts, aiming to clarify whether SPT should be considered a contraindication to this minimally invasive curative approach.

## 2 Materials and methods

This was a single-center, retrospective cohort study conducted at the Department of General Surgery I, Sixth People's Hospital of Shenyang. Clinical data were collected from patients with early HCC who underwent ultrasound-guided laparoscopic microwave ablation (MWA) between February 2023 and February 2024. The study was approved by the Ethics Committee of our hospital, and the requirement for informed consent was waived due to the retrospective nature of the study.

### 2.1 Patients

We retrospectively reviewed the clinical data of patients who underwent ultrasound-guided laparoscopic MWA in the Department of General Surgery I of our hospital between February 2023 and February 2024.According to preoperative PLT, patients were assigned to either the study group or the control group.

Study group inclusion criteria were as follows: (1) HCC diagnosed as BCLC stage 0/A by two or more imaging modalities prior to surgery; (2) preoperative Child-Pugh liver function grade A or B; (3) receipt of preoperative platelet-increasing therapy with a PLT <50 × 10^9^/L within 3 days before surgery; (4) absence of prior antitumor therapy; and (5) postoperative pathological confirmation of HCC.

Exclusion criteria included: (1) concomitant splenectomy; (2) postoperative pathological findings inconsistent with HCC; (3) preoperative PLT >50 × 10^9^/L after platelet-increasing therapy; (4) alternative causes of TP, such as hematologic disorders; and (5) loss to follow-up.

Control group inclusion criteria were as follows: (1) HCC diagnosed as BCLC stage 0/A by two or more imaging examinations before surgery; (2) preoperative Child-Pugh liver function grade A or B; (3) PLT >150 × 10^9^/L within 3 days prior to surgery; (4) no history of antitumor treatment; and (5) postoperative pathological confirmation of HCC.

Exclusion criteria included: (1) postoperative pathology inconsistent with HCC; and (2) loss to follow-up.

### 2.2 Treatment protocol

#### 2.2.1 Preoperative preparation

Routine laboratory testing and imaging assessments were completed before surgery. For tumors located at the hepatic margin, indocyanine green was administered preoperatively to enable intraoperative staining for accurate localization. Preoperative platelet-increasing therapy included one or more of the following modalities: oral thrombopoietin receptor agonists (e.g., eltrombopag, avatrombopag, or hetrombopag), subcutaneous recombinant human thrombopoietin (rhTPO), subcutaneous recombinant human interleukin-11 (rhIL-11), and platelet transfusion. The specific regimen was selected by the treating physician based on preoperative platelet count, bleeding risk, availability, and patient tolerance. The duration of therapy ranged from 7 to 20 days. For patients in the study group, surgery was performed when the preoperative platelet count remained below 50 × 10^9^/L within 3 days before surgery despite platelet-increasing therapy.

#### 2.2.2 Surgical procedure

The procedure was performed with the patient in the supine position under general anesthesia. A 10-mm longitudinal incision was created below the umbilicus for laparoscopic exploration. Additional 10-mm and 5-mm ports were established below the xiphoid process and along the right midclavicular line at the costal margin, respectively. Artificial ascites was induced by instillation of 500–1,000 mL of 5% glucose solution into the perihepatic space via a suction device. Laparoscopic ultrasound (LUS) was used to examine the entire liver and identify the target lesion, followed by contrast-enhanced ultrasound to further delineate tumor location and size. Fluorescence laparoscopy combined with ultrasound guidance was applied to localize the lesion and determine an optimal puncture trajectory, avoiding the main branches of the Glisson system, the inferior vena cava, the gallbladder, the diaphragm, and other critical structures. Tumor tissue was obtained using a biopsy needle to ensure adequate sampling. Subsequently, under direct laparoscopic visualization with real-time ultrasound guidance, a microwave antenna was advanced into the lesion to initiate ablation. Upon completion of ablation, the needle tract was coagulated before withdrawal. The microwave ablation system used wasKY-2000 microwave ablation system (Kangyou Medical, Nanjing, China, operating at a frequency of 2,450 MHz. A 15-gauge, 18-cm microwave antenna was used. Ablation was performed at a power output of 50W for 5–10 min per cycle. The number of ablation cycles was determined by tumor size and real-time ultrasound findings; typically, 1–3 cycles were applied. Adequate ablation was defined as a hyperechoic ablation zone completely covering the tumor with a safety margin of at least 5 mm beyond the tumor boundary on contrast-enhanced ultrasound, with no residual enhancement in the arterial phase. If residual enhancement was observed, additional ablation cycles were performed until this criterion was met. Upon completion of ablation, the needle tract was coagulated before withdrawal. A second contrast-enhanced ultrasound examination was performed to verify complete ablation of the target lesion. Peritoneal fluid was aspirated, and after confirmation of hemostasis, all port sites were sutured (Figure 1). The procedure was then concluded.

**Figure 1:**
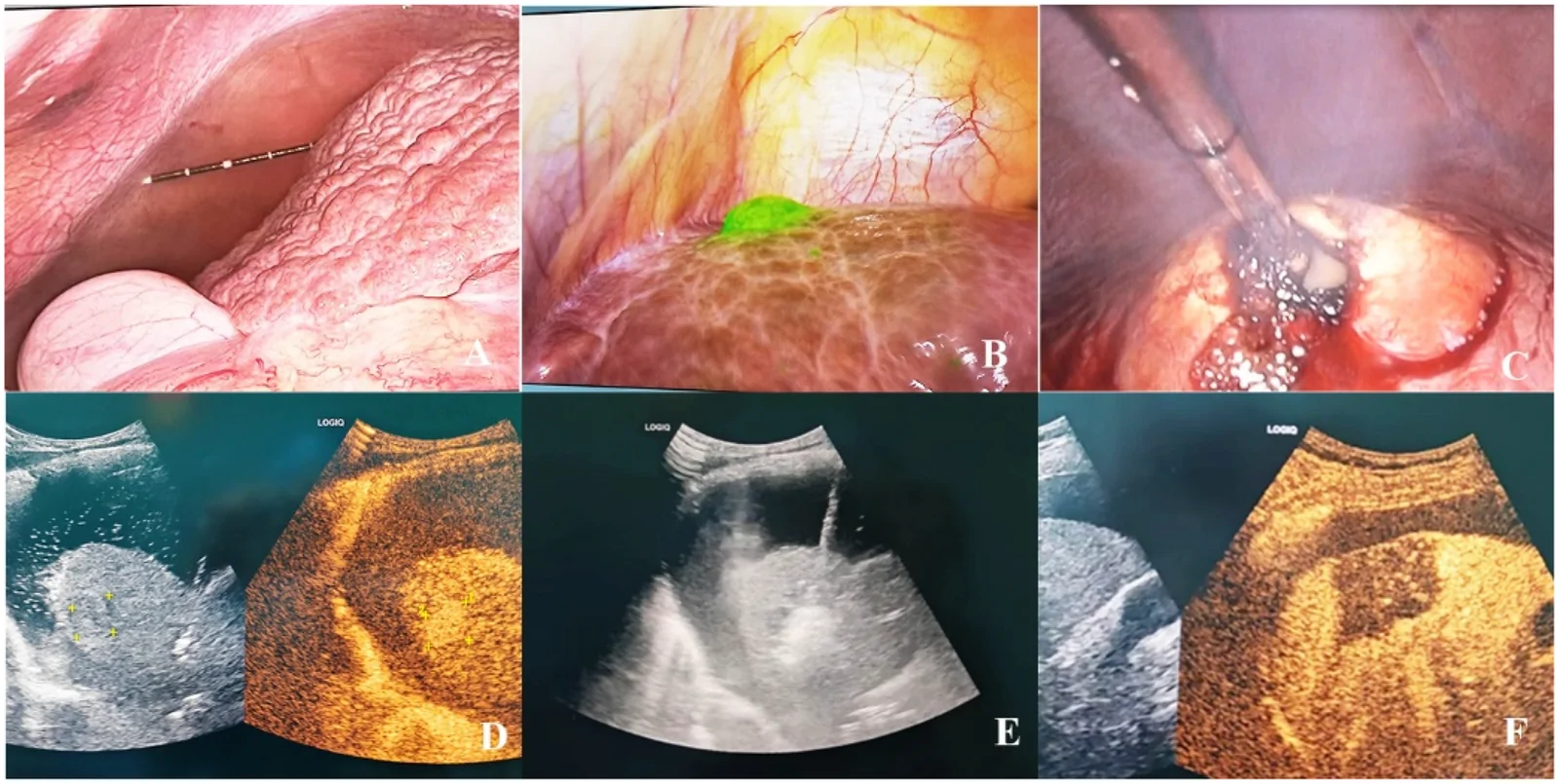
Intraoperative laparoscopic and ultrasound images. **(A)** Isolation and protection achieved by artificial ascites, with direct laparoscopic visualization of the ablation needle. **(B)** Preoperative indocyanine green staining of the tumor on the liver surface. **(C)** Direct laparoscopic visualization and ablation of a lesion located on the surface of the left hepatic lobe. **(D)** Preoperative contrast-enhanced ultrasound imaging of the target tumor with measurement of tumor size. **(E)** Real-time percutaneous ultrasound monitoring during ablation. **(F)** Following ablation, contrast-enhanced ultrasound was repeated to confirm an adequate ablation margin; the post-ablation necrotic area is shown.

#### 2.2.3 Postoperative treatment and follow-up

Patients were maintained on fasting and water restriction on the day of surgery and received hemostatic agents, hepatoprotective medications, and parenteral nutrition as supportive therapy. Postoperative evaluations included liver function tests, serum albumin levels, coagulation profiles, complete blood counts, and inflammatory markers, with subsequent management adjusted according to these results. The initial postoperative follow-up was scheduled at one month after discharge, followed by evaluations every three months, including imaging studies (contrast-enhanced CT or MRI of the liver) and laboratory examinations.

### 2.3 Outcome measures

Baseline and perioperative variables were compared between the two groups, including tumor location, tumor size, tumor stage, preoperative liver function classification, operative duration, estimated intraoperative blood loss, length of postoperative hospitalization, occurrence of postoperative complications (including fever, infection, pleural effusion, ascites, liver failure, postoperative hemorrhage, and related events), and disease-free survival.

### 2.4 Ethics

This study was approved by the Ethics Committee of Sixth People's Hospital of Shenyang (ethics number: 2024-10-001-01).

### 2.5 Statistical methods

Propensity score matching (PSM) was applied to adjust for baseline differences between the study and control groups, thereby approximating the conditions of a randomized controlled trial and improving the reliability of the conclusions. Propensity scores were calculated for each patient using multivariate logistic regression based on age, sex, BMI, Child-Pugh score, and tumor diameter stage. Matching was performed at a 1:1 ratio with a caliper width of 0.2. Statistical analyses were conducted using SPSS 26. Continuous variables with normal distributions are expressed as mean ± standard deviation and were compared using the independent-samples t test or the Mann–Whitney U test, as appropriate. Categorical variables were compared using the chi-square test or Fisher's exact test. Disease-free survival was evaluated using Kaplan–Meier analysis, and GraphPad Prism was used to generate disease-free survival curves. A *P* value < 0.05 was considered statistically significant.

## 3 Results

### 3.1 Patient baseline characteristics

A total of 97 patients were enrolled, including 49 in the study group and 48 in the control group. After PSM, 22 patients were retained in each group (Figure 2). The PLT was (38.6 ± 7.17) × 10^9^/L in the study group and (182.7 ± 26.65) × 10^9^/L in the control group. Prior to PSM, statistically significant differences were observed in preoperative Child-Pugh liver function classification and sex distribution between the two groups (*P* = 0.01), whereas no significant differences were identified in other baseline variables (Table 1). Following matching, all baseline characteristics were well balanced between the two groups (Table 2).

**Figure 2:**
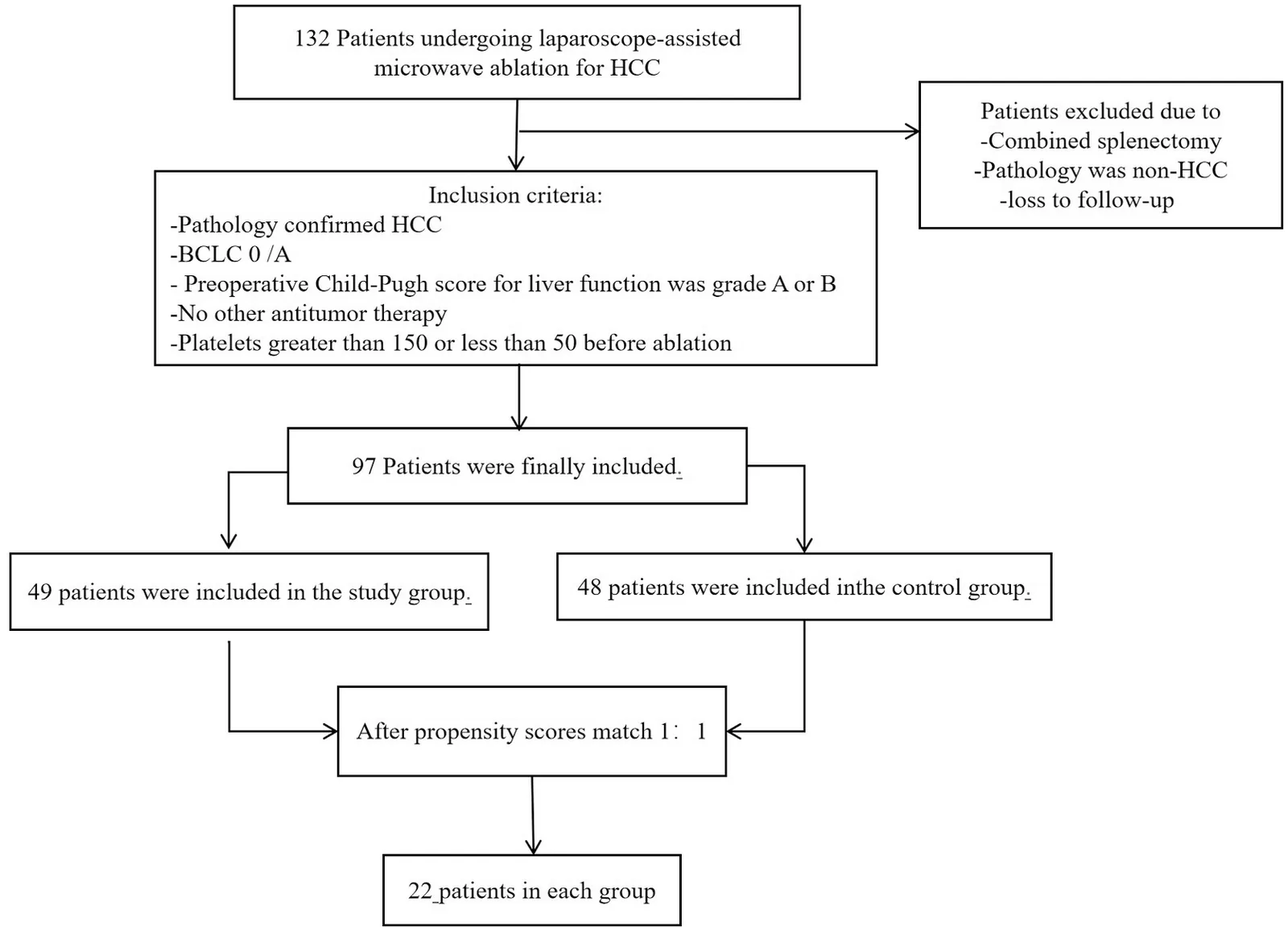
Patient inclusion and matching flowchart.

**Figure 3:**
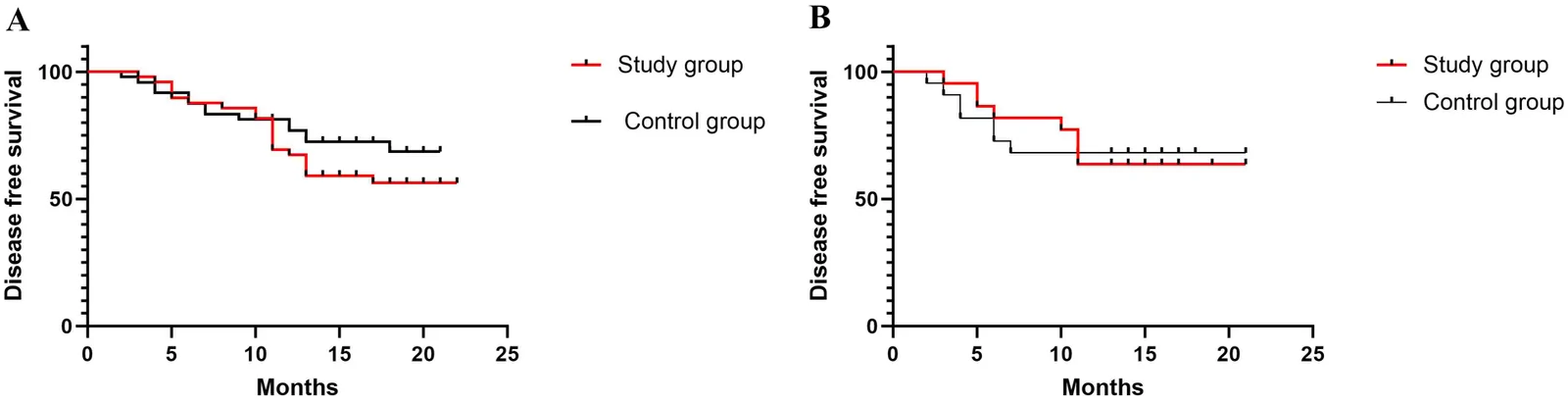
Comparison of disease-free survival between the two groups, with red indicating the study group and black indicating the control group. **(A)** No statistically significant difference was observed between groups before PSM. **(B)** No statistically significant difference was observed between groups after PSM.

**Table 1:** Baseline characteristics of patients before PSM.

Characteristics	Severe TP group （*n* = 49)	Normal PLT group （*n* = 48)	*p-value*
Age (years)	58.22 ± 7.7	60.44 ± 11.4	0.265
Sex (male/female)	27/22	36/12	0.040*
BMI	22.5 ± 3.4	22.9 ± 3.1	0.367
Chronic liver disease（HBV/HCV/alcoholic liver disease）	34/13/2	35/10/3	0.742
PLT (10^9^/L）	38.6 ± 7.17	182.7 ± 26.65	＜0.01*
Child-Pugh grade（A/B）	32/17	47/1	＜0.01*
BCLC 0/A	40/9	38/10	0.760
Tumor location（Caudate lobe/Left liver/Right liver/Left and right liver）	1/11/35/2	2/9/34/3	0.864
Tumor diameter (cm)	2.04 ± 0.99	1.95 ± 0.93	0.625

**Table 2:** Baseline characteristics of patients after PSM.

Characteristics	Severe TP group （*n* = 22)	Normal PLT group （*n* = 22)	*p-value*
Age (years)	58.73 ± 8.27	58.77 ± 11.98	0.988
Sex (male/female)	12/10	13/9	0.761
BMI	21.36 ± 3.8	21.97 ± 3.2	0.745
chronic liver disease（HBV/HCV/alcoholic liver disease）	16/6/0	19/2/1	0.196
Child-Pugh grade（A/B）	21/1	21/1	1
ALBI score	−2.324	−3.225	0.000307
TBIL μmol/L	23.1	9.35	<0.001
ALB g/L	36.8	46.1	0.0048
INR	1.205	1.055	<0.001
BCLC 0/A	17/5	19/3	0.434
Tumor location（Caudate lobe/Left liver/Right liver/Left and right liver）	1/6/14/1	2/4/15/1	0.857
Tumor diameter (cm)	2.00 ± 0.95	2.13 ± 0.86	0.627

### 3.2 Preoperative platelet-increasing therapy and platelet trends

All 49 patients in the study group received preoperative platelet-increasing therapy. The modalities included oral thrombopoietin receptor agonists (TPO-RAs; *n* = 41), subcutaneous recombinant human interleukin-11 (rhIL-11; *n* = 10), and platelet transfusion (*n* = 26). Combined therapy was administered in 21 patients. The median duration of therapy was 11 days (range 7–20). The platelet count before platelet-increasing therapy was 32.5 ± 9.13 ×  10^9^/L; immediately before surgery, it was 38.6 ± 7.17 × 10^9^/L; on postoperative day 1, it was 24.1 ± 7.2 × 10^9^/L; and on postoperative day 7, it was 34.8 ± 7.35 × 10^9^/L. No thrombotic events, including portal vein thrombosis, deep vein thrombosis, or pulmonary embolism, were observed in either group during the perioperative period or follow-up.

### 3.3 Perioperative variables and follow-up results

Intraoperative blood loss was significantly greater in the study group than in the control group both before and after PSM. However, no patient in either group required intraoperative or postoperative blood transfusion. No hemoperitoneum requiring intervention, puncture tract bleeding, conversion to open surgery, reoperation, ICU admission, bile leak, intra-abdominal infection, ascites, pleural effusion, or readmission within 30 days occurred in either group. In the pre-matching cohort, one patient in the study group developed postoperative liver failure, which resolved after two sessions of artificial liver therapy; no postoperative liver failure occurred in the post-matching cohort. No perioperative deaths occurred in either group. These results are summarized in Table 3. Postoperative pain was defined as pain requiring analgesic treatment, as documented in the nursing records. The incidence of postoperative pain requiring analgesia was 36.7% in the study group and 28.9% in the control group before PSM (*P* = 0.564), and 46.7% versus 31.4% after PSM (*P* = 0.536).

**Table 3:** Comparison of perioperative conditions between two groups.

Variables	Before PSM		*p-value*	After PSM		*p-value*
	Severe TP group (*n* = 49)	Normal PLT group (*n* = 48)		Severe TP group (*n* = 22)	Normal PLT group (*n* = 22)	
Operation time (min)	86.08 ± 25.05	88.46 ± 26.85	0.674	89.01 ± 23.04	88.72 ± 24.62	0.279
Intraoperative blood loss (mL)	45.69 ± 8.5	19.71 ± 6.37	＜0.01*	42.8 ± 4.33	20.03 ± 5.9	＜0.01*
Incidence of postoperative complications (%)	26.53	20.83	0.509	22.72	18.18	0.709
Pain	14.28	10.42		13.64	13.64	
Infection	2.04	4.17		0	0	
Pleural/Peritoneal Effusion	8.16	6.25		9.1	4.54	
Hypohepatia	2.04	0		0	0	
Postoperative hospital stay (day)	7.53 ± 2.45	8.02 ± 3.06	0.386	7.64 ± 2.8	7.98 ± 1.94	0.277

Follow-up was completed in February 2025, with a duration ranging from 3 to 22 months. No deaths were recorded in either group during the follow-up period. The median follow-up duration, estimated using the reverse Kaplan–Meier method, was 11.0 months. All 97 patients included in the analysis had evaluable follow-up data. Patients without a DFS event were censored at their last available follow-up. Before PSM, 13 DFS events occurred in the severe thrombocytopenia group and 9 in the normal platelet group. The median DFS was not reached in either group. The 12-month DFS rates were 71.0% (95% CI, 54.2%–82.6%) and 76.0% (95% CI, 57.4%–87.3%), respectively, with no significant difference between the groups (log-rank *P* = 0.209). After PSM, the median DFS remained unreached in both groups. The 12-month DFS rates were 70.3% (95% CI, 40.8%–87.1%) in the severe thrombocytopenia group and 83.1% (95% CI, 61.0%–93.3%) in the normal platelet group, with no significant difference between the groups (log-rank *P* = 0.835).

## 4 Discussion

In this retrospective cohort study, we evaluated the safety and short-term efficacy of ultrasound-guided laparoscopic microwave ablation (MWA) in patients with early HCC and severe thrombocytopenia. The principal findings were as follows: (1) intraoperative blood loss was significantly greater in the severe thrombocytopenia group than in the normal platelet group, both before and after propensity score matching (PSM); (2) operative duration, length of postoperative hospitalization, and the incidence of postoperative complications did not differ significantly between the two groups; and (3) no statistically significant difference in disease-free survival was observed. These findings suggest that although severe thrombocytopenia increases intraoperative bleeding risk, it does not necessarily translate into worse short-term postoperative recovery or early oncologic outcomes when laparoscopic MWA is performed with careful hemostatic precautions.

The greater intraoperative blood loss observed in the severe thrombocytopenia group is biologically plausible. Patients with cirrhosis and severe thrombocytopenia frequently have portal hypertension, hypersplenism, splenomegaly, and abdominal wall varices, all of which can increase bleeding during pneumoperitoneum establishment, liver puncture, and ablation. In addition, thrombocytopenia may coexist with impaired platelet function and an altered coagulation balance in cirrhosis. Nevertheless, several technical measures may have mitigated the clinical consequences of this bleeding risk. Artificial ascites helped separate adjacent organs, direct laparoscopic visualization allowed prompt identification and control of bleeding, and prolongation of needle-tract coagulation by 20–30 s may have reduced puncture-site bleeding. These factors may explain why the higher intraoperative blood loss did not translate into higher complication rates or longer hospitalization. However, given the limited sample size, particularly after PSM, and the short follow-up duration, these findings should be interpreted with caution. Larger prospective studies are needed to confirm whether severe thrombocytopenia truly has no meaningful impact on postoperative recovery and long-term outcomes.

These observations are consistent with previous studies suggesting that local thermal ablation may be feasible in patients with cirrhosis and severe thrombocytopenia (8). However, most prior studies have focused on percutaneous ablation or have stratified patients by platelet count rather than comparing laparoscopic MWA with normal platelet controls. Our study therefore adds to the literature by specifically evaluating laparoscopic MWA in early HCC patients with severe thrombocytopenia.

Liver cirrhosis is the advanced stage of chronic liver disease, and 70%–78% of patients with cirrhosis develop hepatopathy-related thrombocytopenia (HRT) due to portal hypertension, hypersplenism, and reduced hepatic thrombopoietin synthesis; because more than 80% of HCC arises in cirrhotic livers, thrombocytopenia is a major challenge in HCC management (18, 19). The severity of thrombocytopenia generally reflects the extent of cirrhosis rather than tumor stage, and a platelet count <50 × 10^9^/L is considered a high-risk condition for hepatic surgery.

Current approaches for correcting HRT include pharmacologic therapy or platelet transfusion, splenectomy, and splenic embolization. Pharmacologic agents may produce modest increases in PLT, whereas platelet transfusion achieves a rapid but transient effect and may induce antibody formation, reducing the efficacy of subsequent transfusions (20). In the present study, patients received preoperative platelet-increasing therapy, including oral thrombopoietic agents, subcutaneous administration of recombinant human thrombopoietin, recombinant human interleukin-11, and platelet transfusions, for durations ranging from 7 to 20 days. Clinical observations indicate that in patients with PLT below 30 × 10^9^/L, conservative measures frequently fail to elevate platelet levels above 50 × 10^9^/L within a short timeframe.

Our findings are broadly consistent with previous studies evaluating local thermal ablation in thrombocytopenic patients with HCC. Xiao et al. retrospectively analyzed 166 cirrhotic HCC patients who underwent radiofrequency ablation (RFA) and found that perioperative major hemorrhage occurred in only 2.4% of patients, with comparable postoperative complication rates across platelet strata (≤ 30 × 10^9^/L, 31–49 × 10^9^/L, and ≥ 50 × 10^9^/L) (21). Similarly, Zhao et al. reported an overall bleeding complication rate of 4.4% after local thermal ablation in 473 cirrhotic HCC patients, with no significant difference among patients with platelet counts > 50 × 10^9^/L, 30–50 × 10^9^/L, and ≤ 30 × 10^9^/L (3.9% vs. 6.4% vs. 3.3%, *P* = 0.410) (8). These findings support the notion that severe thrombocytopenia does not inevitably lead to a substantial increase in clinically significant bleeding complications after thermal ablation, which aligns with the absence of significant differences in complication rates observed in our cohort. However, it should be noted that in our study, intraoperative blood loss was significantly greater in the severe thrombocytopenia group, whereas the cited studies primarily focused on postoperative bleeding complications. This discrepancy may reflect differences in outcome definitions: intraoperative blood loss is a continuous measure sensitive to even modest increases in bleeding, whereas clinically significant postoperative bleeding complications represent a higher threshold. In addition, our study specifically evaluated laparoscopic MWA, whereas the aforementioned studies predominantly assessed percutaneous RFA or mixed ablation modalities. The laparoscopic approach allows direct visualization and prompt hemostasis, which may have mitigated the clinical impact of increased intraoperative bleeding.

For HCC patients with STP, concurrent splenectomy during tumor treatment or preoperative splenic embolization has been proposed to improve clinical outcomes (12, 22). As HCC advances, additional therapeutic modalities such as surgical resection, interventional procedures, and targeted immunotherapy may be required, with TP persisting as a major risk factor. Splenectomy can rapidly elevate PLT in the short term; however, it does not reduce intraoperative bleeding risk and is associated with an increased incidence of postoperative infection. Portal vein thrombosis, particularly extensive mesenteric venous thrombosis, is associated with unfavorable prognosis (23, 24). Although splenic embolization is a minimally invasive intervention, it is also associated with complications including splenic ischemia and necrosis, abscess formation, and splenic rupture (25). In addition, several molecular studies have indicated that elevated PLT (>450 × 10^9^/L) may correlate with poor oncologic outcomes through modulation of the immune and tumor microenvironment (26–30).

With respect to therapeutic strategies for early HCC, liver transplantation can concurrently treat cirrhosis and HCC, although widespread application remains limited. Partial hepatectomy is generally regarded as the preferred curative approach; however, patients with severe hypersplenism often experience suboptimal short-term outcomes (31). Consequently, local ablation represents a suitable alternative for this patient population (13, 32–34). This approach is characterized by minimal invasiveness, rapid postoperative recovery, and repeatability, and has become a commonly applied modality for early HCC. Compared with radiofrequency ablation, MWA provides a larger ablation zone, shorter ablation time, and reduced susceptibility to the heat-sink effect, and is generally considered to offer technical advantages (35, 36).

The preference for laparoscopic MWA over percutaneous ablation in patients with severe thrombocytopenia and cirrhosis is based on three considerations. First, patients with severe thrombocytopenia are at increased risk of bleeding, and the laparoscopic approach allows direct visualization of the ablation needle, tumor, and surrounding structures throughout the procedure. Bleeding from the puncture site or liver surface can therefore be promptly identified and controlled, and needle-tract coagulation can be prolonged by 20–30 s compared with patients with normal platelet counts. In contrast, percutaneous ablation relies on indirect ultrasound monitoring, which may delay recognition of bleeding. Second, artificial ascites can be created under laparoscopic guidance to separate the liver from the diaphragm, gallbladder, gastrointestinal tract, and abdominal wall, thereby reducing the risk of thermal injury to adjacent organs and enabling safe ablation of lesions in difficult locations. This is particularly relevant because percutaneous puncture of superficial lesions carries a risk of tract seeding, and lesions adjacent to the diaphragm may be obscured by pulmonary air. Third, comparative evidence suggests that laparoscopic MWA may achieve better oncologic outcomes than percutaneous MWA. In a comparative analysis of 275 patients, laparoscopic MWA was associated with significantly lower local recurrence (5% vs. 22%, *P* = 0.002) and longer median recurrence-free survival (15.8 months vs. 5.6 months, *P* = 0.0002) than percutaneous MWA (37). These advantages are particularly relevant in patients with severe thrombocytopenia, in whom percutaneous ablation carries a higher risk of bleeding and incomplete ablation.

During the procedure, several technical considerations were identified. Patients with STP frequently present with splenomegaly and varices of the abdominal wall and periumbilical region. In individuals with grade III splenomegaly, the spleen may extend below the umbilicus or even beyond the right side of the abdominal midline. During establishment of pneumoperitoneum, the puncture site for the insufflation needle should avoid the spleen and varicose veins, and an open pneumoperitoneum technique may be required in selected cases. Some patients have a history of spontaneous bacterial peritonitis with subsequent intra-abdominal adhesions, necessitating adhesiolysis as an initial step. In addition, the maximum tumor diameter observed on contrast-enhanced ultrasound is often slightly larger than that measured on enhanced CT or MRI; therefore, the ablation zone should be determined according to the maximal diameter identified on contrast ultrasound to ensure an adequate safety margin. Following ablation, the duration of needle withdrawal and tract coagulation should be prolonged by 20–30 s compared with patients with normal PLT to reduce the risk of puncture-site bleeding. Although greater intraoperative blood loss was observed in the STP group, no statistically significant differences were noted in operative duration, length of postoperative hospitalization, or complication rates. Given the limited sample size, particularly after PSM, and the short follow-up, these findings should be regarded as preliminary.

These findings have several potential clinical implications. First, severe thrombocytopenia should not be regarded as an absolute contraindication to curative local ablation in patients with early HCC, provided that appropriate preoperative platelet-increasing therapy is administered and meticulous intraoperative hemostatic precautions are taken. The laparoscopic approach offers specific advantages in this setting, including direct visualization of the ablation needle and surrounding structures, the ability to create artificial ascites to separate adjacent organs, and the capacity for prompt identification and control of bleeding. Second, our results support the use of laparoscopic MWA as a viable treatment option for early HCC patients with severe thrombocytopenia who are not candidates for surgical resection because of hypersplenism and impaired liver function. Third, the significantly greater intraoperative blood loss observed in the severe thrombocytopenia group underscores the need for careful preoperative optimization of platelet counts and a low threshold for intraoperative hemostatic measures. Finally, the absence of significant differences in postoperative complications and disease-free survival suggests that, with appropriate perioperative management, the short-term oncologic outcomes of laparoscopic MWA are not compromised by severe thrombocytopenia. Nevertheless, these findings should be confirmed in larger prospective studies before they are incorporated into clinical guidelines.

This study has several limitations. First, it was a single-center retrospective study with a relatively small sample size, particularly after PSM (22 patients per group), which limits statistical power. Second, follow-up was relatively short, and disease-free survival data were censored at the last imaging follow-up; long-term oncologic outcomes therefore remain uncertain. Second, we excluded patients with moderate thrombocytopenia (PLT 50–150 × 10^9^/L), as this subgroup does not typically require preoperative platelet-increasing therapy and was not the focus of the present investigation. However, this exclusion precludes analysis of a potential dose-response relationship between platelet count and perioperative outcomes and limits the generalizability of our findings to the broader thrombocytopenic population. Third, preoperative platelet-increasing therapy was heterogeneous in terms of modality, dose, and duration, which may have influenced perioperative outcomes. Finally, although PSM was used to balance baseline characteristics, residual confounding cannot be excluded. Multicenter prospective studies with larger sample sizes and longer follow-up are needed to confirm our findings.

## 5 Conclusions

In this single-center retrospective cohort study, ultrasound-guided laparoscopic MWA was performed in patients with early HCC and severe thrombocytopenia with acceptable short-term outcomes. Although intraoperative blood loss was greater in the severe thrombocytopenia group, no statistically significant differences were observed in operative duration, length of postoperative hospitalization, postoperative complication rates, or disease-free survival compared with patients with normal platelet counts. These findings suggest that laparoscopic MWA may be considered a feasible treatment option for selected patients with early HCC and severe thrombocytopenia. However, given the retrospective design, relatively small sample size (particularly after propensity score matching), short follow-up duration, and the single-center setting, these results should be regarded as preliminary. Multicenter prospective studies with larger sample sizes and longer follow-up are warranted to confirm the safety and long-term oncologic efficacy of laparoscopic MWA in this high-risk population.

## Data Availability

The raw data supporting the conclusions of this article will be made available by the authors, without undue reservation.
